# Microbial mats: an ecological niche for fungi

**DOI:** 10.3389/fmicb.2012.00424

**Published:** 2013-04-05

**Authors:** Sharon A. Cantrell, Lisabeth Duval-Pérez

**Affiliations:** Department of Biology, School of Science and Technology, Universidad del TuraboGurabo, PR, USA

**Keywords:** Caribbean, tropical, hypersaline, ITS region, EPS, *Acremonium*, *Aspergillus*, *Cladosporium*

## Abstract

Fungi were documented in tropical hypersaline microbial mats and their role in the degradation of complex carbohydrates (exopolymeric substance – EPS) was explored. Fungal diversity is higher during the wet season with *Acremonium*, *Aspergillus*, *Cladosporium*, and *Penicillium* among the more common genera. Diversity is also higher in the oxic layer and in young and transient mats. Enrichments with xanthan (a model EPS) show that without antibiotics (full community) degradation is faster than enrichments with antibacterial (fungal community) and antifungal (bacterial community) agents, suggesting that degradation is performed by a consortium of organisms (bacteria and fungi). The combined evidence from all experiments indicates that bacteria carried out approximately two-third of the xanthan degradation. The pattern of degradation is similar between seasons and layers but degradation is faster in enrichments from the wet season. The research suggests that fungi thrive in these hypersaline consortia and may participate in the carbon cycle through the degradation of complex carbohydrates.

## INTRODUCTION

Cantrell et al. (2011) reviewed unusual fungal niches of which most are considered extreme environments including Antarctic dry valleys, deep sea sediments, hydrothermal vents, microbial mats, and salterns. All of these environments have been extensively studied for Eubacteria and Archaea, while a few studies have included Eukarya (Gunde-Cimerman et al., 2000, 2004; Edgcomb et al., 2002; Fell et al., 2006; Feazel et al., 2008; Alexander et al., 2009). Eukarya includes a metabolic diverse community of autotrophic, heterotrophic, and mixotrophic members of Cercomonads, Chlorophyte, Choanoflagellida, Ciliophora, Fungi, Radiolaria, Stramenopila, and Metazoa. Because both oxic and anoxic conditions are present in many unusual and extreme environments, they can harbor eukaryotic organisms that are strict aerobes as well as those that are facultative anaerobes and strict anaerobes. Fungi have been documented in all these environments representing between 2 and 20% of the total eukaryal community and represented by members of the Ascomycota, Basidiomycota, and Chytridiomycota.

**FIGURE 1:**
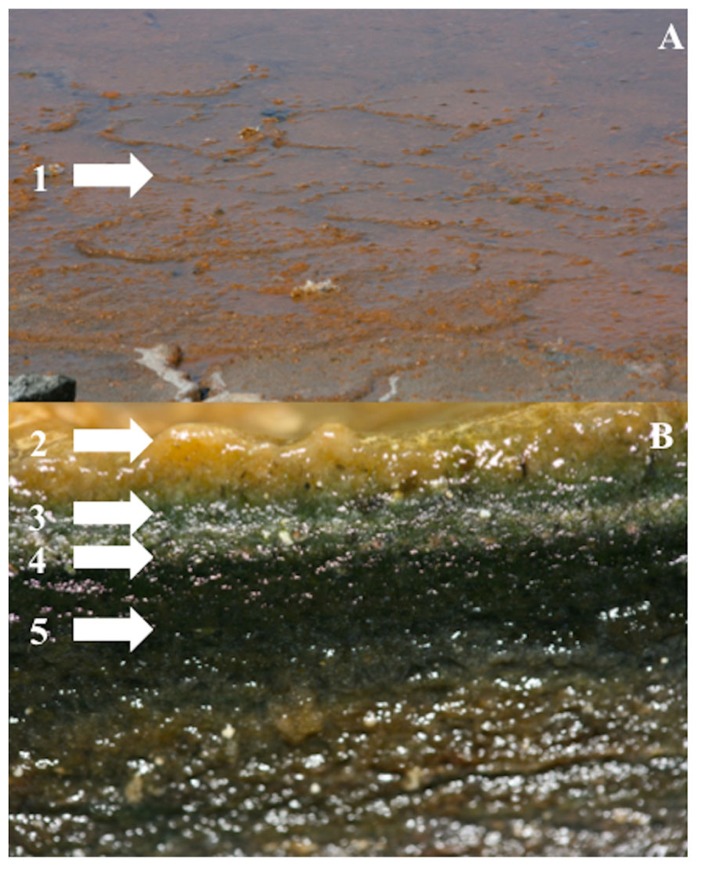
**Non-lythifying mature microbial mat from Puerto Rico.** **(A)** View of the surface of the mat cover with an orange slime that correspond to the EPS. **(B)** Cross section showing the laminated structure of mat. (1–2) EPS, (3) First layer (0–1 mm), (4) Second layer (1–2 mm), (5) >2 mm (Photos by S. A. Cantrell).

Microbial mats are self-sustained vertically laminated, organo-sedimentary structures developing on solid surfaces (**Figure 1**). These can be found in a wide variety of ecosystems from marine intertidal and subtidal zones, fresh water rivers and even extreme environments such as hypersaline ponds, evaporation salterns, and hot springs (Awramik, 1984; Castenholtz, 1984; Anderson et al., 1987; Casillas-Martínez et al., 2005; Gerdes, 2007). Mats can be lithifying or non-lithifying, which means that when the precipitation of minerals exceeds dissolution they can convert loose sediments into rock. Stromatolites are microbial mats and one of the oldest fossils found on Earth dating to 3.4 billion years ago (Allwood et al., 2009). Mats have earned the name “complex biofilms” because their development on physical substrates also occurs as a sequence of processes. Fluctuating diel and seasonal physicochemical gradients characterize these organo-sedimentary ecosystems resulting in both strata and microenvironments that harbor specific microbial communities (Dupraz and Visscher, 2005; Visscher and Stolz, 2005).

There is a physical conditioning of the surface with the deposition of substances that attract and permit the adherence of bacteria. After initial colonization, the bacteria form a thin subsurface layer which thickens as they reproduce and new bacteria arrive, establishing a community that consists of multiple populations (Castenholtz, 1984; Atlas and Bartha, 1997). Primary production occurs in the uppermost layer that corresponds to the oxic zone, fueling heterotrophic activity in the entire mat. Oxygen concentration decreases with depth and the mat is anaerobic below 2 mm. The populations form a consortium as defined in Paerl (2000): several species or populations of microorganisms function in a coordinated, complementary fashion, so that production, growth, and nutrient cycling are enhanced over what a single species or population can achieve alone under similar environmental conditions.

Members of microbial mat communities have been arranged into approximately seven biogeochemical/trophic categories: (1) photolithoautotrophs (i.e., cyanobacteria); (2) aerobic (chemoorgano-) heterotrophs; (3) fermenters; (4) anaerobic heterotrophs (sulfate-reducing bacteria; SRB); (5) sulfide oxidizing bacteria (SOB); (6) anoxyphototrophs [i.e., purple and green (non) sulfur bacteria]; and (7) methanogens (van Gemerden, 1993; Dupraz and Visscher, 2005). Molecular research has shown that there are many eukaryotic groups inhabiting these ecosystems including algae, ciliates, flagellates, fungi, and nematodes (Cantrell et al., 2006; Feazel et al., 2008). Recent studies in hypersaline cyanobacterial mats have revealed a potential to be used as indicators of elevated hurricane activity and their relation to climate change (Paerl et al., 2003). This study concludes that hypersaline mats can be excellent indicators of short- and long-term climate changes by studying the changes in CO_2_ sequestration, enhanced nutrient cycling, and diversification of the microbial communities. A wide variety of mats have been studied for their diversity and the potential of finding new organisms with useful capabilities (i.e., biotechnological applications). Some of the formations being investigated are in the Bahamas (Baumgartner et al., 2009), Guerrero Negro in Mexico (Spear et al., 2003; Ley et al., 2006), Yellowstone National Park (Ward et al., 1998), the Sečovlje Salterns in Slovenia (Tkavc et al., 2011), the Iberian Peninsula of Spain (Esteve et al., 1992), Lagoa Vermelha in Brazil (Vasconcelos et al., 2006), Sinai (Egypt; Teske et al., 1998), and Cabo Rojo in Puerto Rico (Casillas-Martínez et al., 2005; Cantrell et al., 2006).

High salinity makes an extreme environment for most organisms and reports of fungal diversity from such habitats began around 1999 (Gunde-Cimerman et al., 2000, 2004). Hypersaline microbial mats, dominated by Eubacteria and Archaea, are an unusual niche for fungi due to harsh conditions of high salinity, low oxygen, and high H_2_S concentrations. Microbial mat communities have been extensively studied for decades and even though the presence of fungal communities was speculated (van Gemerden, 1993; Gerdes, 2007) studies to characterize the group were not performed before 2006. Fungi have been reported to occur in hypersaline microbial mats in Mexico, Australia, and Puerto Rico (Cantrell et al., 2006; Feazel et al., 2008; Allen et al., 2009; Cantrell and Báez-Félix, 2010). Both Feazel et al. (2008) and Allen et al. (2009) used 18S universal eukaryotic primers to document the eukaryotic diversity and reported the presence of fungi within the clone libraries. Feazel et al. (2008) found *Metschnikowia bicuspidata* from a well-developed non-lithifying Guerrero Negro mat in México. Allen et al. (2009) reported *Engyodontium album* from a pustular microbial mat in the hypersaline lagoon of Shark Bay, Australia. Tropical hypersaline microbial mats from the salt flats in Cabo Rojo, Puerto Rico (Cantrell et al., 2006; Cantrell and Báez-Félix, 2010) reported greater diversity of fungi as compared to Allen et al. (2009) and Feazel et al. (2008) because a combination of culturing and molecular methods was used (selective culture media, terminal restriction fragments length polymorphisms – TRFLP, and clone libraries of the ITS region of the ribosomal DNA).

**FIGURE 2:**
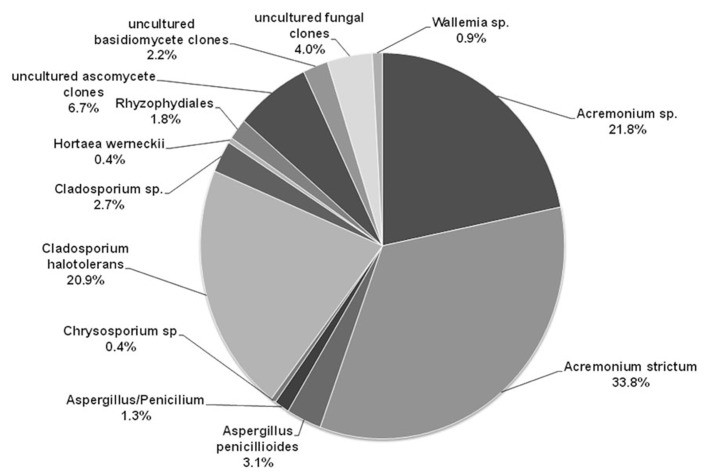
**Diversity of fungi in the mature microbial mat based on clone libraries.** The figure shows the percentage of each fungal species detected within the 225 clones.

Cantrell et al. (2006) reported for the first time melanized filamentous fungi in the genus *Cladosporium*, and non-melanized species in the genera *Penicillium* and *Aspergillus* from tropical transient hypersaline microbial mats of Puerto Rico. Cantrell and Báez-Félix (2010) using TRFLP profiles and clone libraries of the ITS region found that the community of fungi of a mature microbial mat differs between seasons, being more diverse during the rainy season when salinity decreases and oxygen concentrations increase, and that fungal diversity decreases from top (oxic) to the bottom (anoxic) layers of the mats. A recent study using the same combination of methods shows that fungal communities differ between young and transient mats that form only during the rainy season versus mature and well-developed mats that are continuously inundated (Cantrell et al., 2013). Young and transient mats have greater diversity than mature and well-developed mats. So far, a total of 43 species of fungi have been identified from young and mature microbial mats in Puerto Rico, of which 10 are only known from clone libraries. Nine *Aspergillus* and three *Cladosporium* species are known only from cultural studies, with *Aspergillus niger* and *Cladosporium dominicanum* the more frequent species. Based on clone libraries of the ITS region, the fungal community is dominated by *Acremonium strictum* and *Cladosporium halotolerans*, which were not isolated in pure culture in this study (**Figure 2**).

Fungi decompose complex carbohydrates such as lignin, cellulose, and hemicellulose into simpler compounds (i.e., low molecular weight compounds) that are then used by other organisms, thus promoting nutrient recycling. Much investigation has been performed to understand the mechanisms by which fungi decompose complex matter in different terrestrial ecosystems such as tropical forests (Lodge et al., 1996; Green and Highley, 1997). Detritus decomposition is also well understood in coastal marine ecosystems (Fell and Master, 1980; Fell et al., 1984; Acevedo, 1987, 2001; Calzada, 1988; Schmit and Shearer, 2003; Nieves-Rivera, 2005; Kathiresan et al., 2011). Fungi play an important role in decomposing detritus in mangrove forests, including species of *Alternaria*, *Aspergillus*, *Cladosporium*, *Cylindrocarpon*, *Cryptococcus*, *Drechslera*, *Fusarium*, *Geotrichum*, *Gliocladium*, *Gloeosporium*, *Lulworthia*, *Nigrospora*, *Pestalotia*, *Phyllosticta*, *Pichia*, *Rhodotorula*, and *Trichoderma* which have been identified at various stages of leaf litter decomposition (Fell and Master, 1980; Fell et al., 1984; Schmit and Shearer, 2003; Kathiresan et al., 2011). Acevedo (2001) studied the potential role of marine fungi in biotransformation of polycyclic aromatic hydrocarbons. González et al. (2003) reported species of arenicolous fungi for Cuba, including species of *Arenariomyces* and *Corollospora*. Burgaud et al. (2010) reported yeast species belonging to *Candida*, *Cryptococcus*, *Debaryomyces*, *Hortaea*, *Sphaerotheca*, *Pichia*, and *Rhodotorula* from hydrothermal vents. In this paper, we explore microbial mats as an unusual niche for fungi analyzing their potential role in degradation of exopolymeric substances (EPS) which are complex carbohydrates found in these ecosystems.

## MATERIALS AND METHODS

Since fungi play an important environmental function as degraders of complex carbohydrates, the role of fungi in the degradation of EPS and, the communities and individuals carrying out the process was evaluated by Duval-Pérez (2010). Two experiments were performed using xanthan gum and different antibiotics to inhibit certain members of the microbial community. The first experiment was conducted with samples from the dry season and two treatments (full community – no antibiotics and fungal community – four antibacterial agents). The second experiment was conducted with samples from the wet season and three treatments (full community – no antibiotics, bacterial community – antifungal agent and fungal community – four antibacterial agents). Samples were obtained from a mature microbial mat that was producing large quantities of EPS visible as an orange slime over the surface of the mat during the dry season (**Figure 1**). Samples were retrieved by cutting 10 cm ×10 cm squares and dividing it into two layers (top 0–1 mm and bottom 2–20 mm). Mat slurries were prepared from 10 g of each of the mat layers homogenized in 90 ml of a 5% NaCl solution. Duplicate enrichment cultures were prepared by mixing 10 ml of mat slurry with 90 ml of medium (site sea water, 0.1% yeast extract, and 0.25% xanthan gum). Three treatments were monitored: full community (no antibiotics), bacterial community (antifungal Lamisil^®^, 0.1 mg/ml) and fungal community (a mixture of four antibacterial – streptomycin, 1 mg/ml; kanamycin, 0.5 mg/ml; penicillin, 1 mg/ml; and chloramphenicol, 0.1 mg/ml). Cultures were incubated in flasks on a shaker to provide sufficient oxygen at 30°C to mimic the natural temperature conditions. Samples from enrichments were taken weekly to analyze for xanthan concentration for a total of 10 weeks. Xanthan concentration was analyzed using the phenol–sulfuric acid assay in which the glycosidic bond is hydrolyzed releasing a reduced sugar that reacts with phenol to form a yellow pigment that can be detected with a spectrophotometer (Spectronic^TM^Genesys 20 Vis, from Thermo Fisher Scientific; Waltham, MA, USA) at 490 nm (Dubois et al., 1956; Braissant et al., 2009). Independent *t*-test and one-way ANOVA were performed to test the statistical significance between the season, layers, and treatments.

## RESULTS

**FIGURE 3:**
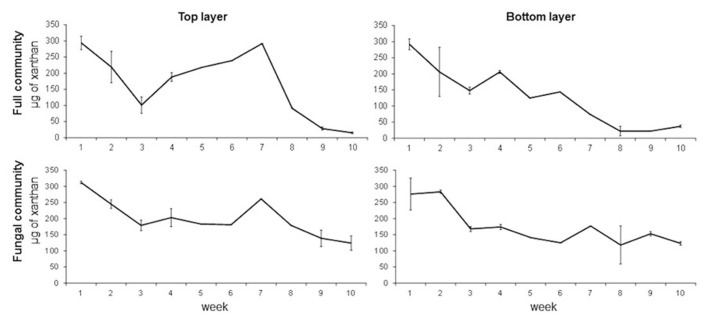
**Time series of xanthan concentration in full and fungal communities’ enrichments from top and bottom layers of a mature microbial mat during the dry season.** Error bars represent the standard deviation of the mean.

The results obtained from the first experiment with samples from the dry season show that xanthan degradation is gradual and constant throughout the 10 weeks in both treatments and layers (**Figure 3**). Enrichments from the full community from the top layer showed a gradual decrease of xanthan concentration during the first 3 weeks with a gradual increase during the next 4 weeks. This observation can be due to the presence of EPS producing microorganisms in the enrichment. After the seventh week, xanthan concentration decreases and by week 9, 90% of the xanthan is degraded. In enrichments from the bottom layer this drastic increase is not observed and by week 8, 92% of the xanthan is degraded. On the other hand, enrichments from the fungal community showed a similar pattern as the full community but less degradation of xanthan is observed. Only 55% of the xanthan is degraded by week 9 and 8 in the top and bottom layers, respectively (**Figure 3**). Differences between layers are only suggestive (*t*-test, *p* = 0.06) but there are significant differences between the treatments (*t*-test, *p* = 0.032).

**FIGURE 4:**
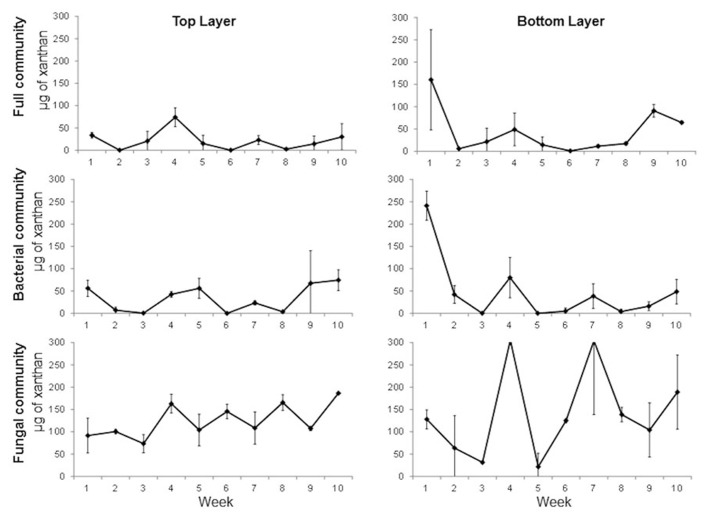
**Time series of xanthan concentration in full, bacterial, and fungal communities’ enrichments from top and bottom layers of a mature microbial mat during the wet season.** Error bars represent the standard deviation of the mean.

Since more degradation was observed in the full versus the fungal communities, a second experiment was performed to include a third treatment to inhibit the fungal community and in which the bacterial community was favored. The experiment was done with samples from the wet season. The results show that xanthan degradation is very fast in the top layer with 90, 78, and 64% degradation in the first week in full, bacterial, and fungal community, respectively (**Figure 4**). The process is delayed by a week in the bottom layer. The same pattern of increasing and decreasing xanthan concentration through time was observed particularly in the enrichment for the fungal community. Significant differences were observed between treatments (one-way ANOVA, *p* = 0.0001) but not between layers (*t*-test, *p* = 0.21). Significant differences were observed between the seasons (*t*-test, *p* = 0.0001). Degradation of xanthan is faster in the enrichments from the wet season in all treatments. This can be an indication that in the dry season the consumption of EPS is slower to maintain protection from external factors, or that the microbial community present during this season does not have the capacity to degrade these compounds.

The combined evidence from all experiments indicates that bacteria carried out approximately two-third of the xanthan degradation. Fungi, however, contribute to the process because degradation is always faster in the enrichments in which the full community is active. The degradation process is not restricted to one layer but occurs throughout the entire microbial mat, which coincides with studies that confirm that EPS concentration decreases with depth and that one of the main factors for this degradation is microbial activity (Green and Highley, 1997; Braissant et al., 2009). *Pichia guilliermondii* and *Penicillium* sp. were two fungal isolates obtained in the fungal enrichment cultures (Duval-Pérez, 2010). Also, TRFLP profiles of the fungal ITS region shows that there are phylotypes that are stimulated in the fungal enrichments which are not seen in the full community and these phylotypes change through time (Duval-Pérez, 2010). Unexpected EPS quantities were observed during the experiment in some of the samples and some of the reasons could be analytical error, the xanthan molecules being affected by external factors such as temperature or more likely, that EPS was produced by the microbial community in the enrichments.

## DISCUSSION

Microbial mats are characterized by a high production of EPS by microbial communities (Decho, 1990, 2000; Braissant et al., 2007, 2009). The EPS provides a cohesive matrix that protects the microbial community from “hostile” environmental conditions (including high UV and salinity) enabling optimal growth, exchange of genetic material, and intra- and interspecies communication. EPS also provides protection from desiccation, a direct effect of solar radiation, and higher production is observed in dry seasons (Sutherland, 2001; Czaczyk and Myszka, 2007; Decho, 2010). Xanthan gum is a model, highly stable EPS that is produced by *Xanthomonas campestris* (Jansson et al., 1975; Holzwarth, 1976; Melton et al., 1976; Sutherland, 1997). Xanthan gum degrading enzymes have been isolated from a salt tolerant *Bacillus *sp., a *Corynebacterium* sp. and *Paenibacillus alginolyticus*, a soil isolate (Cadmus, 1982; Sutherland, 1982, 1984, 1987; Hou et al., 1986; Ruijssenaars et al., 1999). Some fungal cellulases have also been shown to hydrolyse xanthan under restricted conditions (Rinaudo and Milas, 1980; Sutherland, 1984, 1987). The degradation of EPS is believed to contribute in the mineralization of CaCO_3_ (Reid et al., 2000; Dupraz and Visscher, 2005; Ercole, 2007), a very important process in global biogeochemical cycles, and the formation of stromatolites and other microbialites, which represent the oldest evidence of life on Earth (Awramik, 1984; Allwood et al., 2009). However, no organism capable of degrading EPS has been isolated from a microbial mat, although several studies have shown that EPS are readily utilized by the heterotrophic community of a variety of microbial mats, including those in Puerto Rico (Visscher et al., 1998, 1999, 2002, 2010; Decho et al., 2005; Braissant et al., 2009). Also, no other studies have looked at the degradation of EPS using microbial mats slurries and different antibiotic agents as the one presented here.

Microbial mats can be considered relatively simple ecosystems based on the different guilds present but molecular-based studies have shown that mats contain an extremely complex and unique assemblage of microorganisms that interact to produce a highly productive ecosystem surpassing rain forests (Visscher and Stolz, 2005; Baumgartner et al., 2009; Ley et al., 2006). Molecular studies also indicate that eukaryotic organisms (i.e., algae, ciliates, flagellates, fungi, and nematodes) are often present in these ecosystems (Cantrell et al., 2006; Feazel et al., 2008; Cantrell and Báez-Félix, 2010). In this paper, evidence of the diversity and potential role of fungi has been presented. A combination of techniques has been used to document fungal diversity in tropical hypersaline microbial mats. All the techniques were able to detect active and non-active fungal species. Cultural techniques favor fast growing fungi and can obscure the detection of slow growers. Some of the fungi detected may not be true inhabitants of microbial mats, but instead may represent propagules that arrived with wind-blown material or with rain run-off. PCR of environmental DNA usually yields more phylotypes but some groups might not be detected due to biases in DNA extraction, PCR or cloning (Arnold et al., 2007).

Microbial mats are ecosystems where a high recycling of nutrients is observed. The fungal community in the mats utilize organic compounds that are produced within the mat (such as EPS) or that are allochthonous (produced elsewhere). The evidence presented showed that most of the xanthan degradation is done by the bacterial community, though the fungal community may aid in the transformation of xanthan by partially degrading the molecule (Rinaudo and Milas, 1980; Sutherland, 1984, 1987). We showed that mats produce more and degrades less EPS in the dry season than in the wet season, presumably to maintain protection, which is more important during the dry season.The lower diversity and degradation capabilities of the fungal community in the mats could be attributed to low fungal biomass in the samples, absence of natural conditions or DNA extraction and PCR biases (Arnold et al., 2007). A combination of techniques should always be used to overcome possible biases of the different techniques being used. In order to decipher the true fungal community of microbial mats and their potential roles, other techniques that can detect actively growing organisms such as analyzing the transcriptome should be considered.

## Conflict of Interest Statement

The authors declare that the research was conducted in the absence of any commercial or financial relationships that could be construed as a potential conflict of interest.
